# Complete Genome Sequence of *Pithoascus kurdistanensis* CBS 149789, an Endophytic Fungus Isolated from *Papaver bracteatum*

**DOI:** 10.3390/jof11120861

**Published:** 2025-12-05

**Authors:** Sima Mohammadi, Jeff Gauthier, Guillaume Quang Henri Nguyen, Antony T. Vincent, Bahman Bahramnejad, Roger C. Levesque

**Affiliations:** 1 Institut de Biologie Intégrative et des Systèmes, Université Laval, Quebec City, QC G1V 0A6, Canada; sima.mohammadi.1@ulaval.ca (S.M.); jeff.gauthier.1@ulaval.ca (J.G.); guillaume.quang-henri.nguyen.1@ulaval.ca (G.Q.H.N.); antony.vincent@fsaa.ulaval.ca (A.T.V.); 2 Faculté des Sciences de l’Agriculture et de l’Alimentation, Université Laval, Quebec City, QC G1V 0A6, Canada; 3 Department of Plant Production and Genetics, Faculty of Agriculture, University of Kurdistan, Sanandaj 6617715175, Iran; b.bahramnejad@uok.ac.ir

**Keywords:** *Papaver*, Microascaceae, endophytes, genomics, metabolite biosynthesis genes, benzylisoquinoline alkaloids

## Abstract

Endophytic fungi are increasingly recognized as pivotal contributors to plant secondary metabolism, often synthesizing bioactive compounds like those produced by their hosts. We report the first complete genome sequence for *Pithoascus kurdistanensis*, a novel endophyte isolated from *Papaver bracteatum* that produces morphinan alkaloids independently from its host plant. High-quality genomic DNA from *P. kurdistanensis* was subjected to a hybrid sequencing strategy using both Oxford Nanopore long-read and Illumina short-read platforms, yielding a 34.0 Mbp assembly composed of nine chromosomal contigs and four additional minor contigs. This assembly was 97.3% complete as determined by BUSCO and revealed that 6.37% of the genome consists of repetitive elements. A total of 8292 protein-coding genes, including 63 candidate genes potentially involved in isoquinoline alkaloid biosynthesis, have been identified. Phylogenetic analysis based on conserved single-copy orthologs positioned *P. kurdistanensis* within a basal lineage of the Microascaceae. Overall, these results provide foundational insight into the genetic potential of *P. kurdistanensis* as a novel microbial source of morphinan alkaloids, while emphasizing the need for continued functional studies to resolve the underlying biosynthetic pathways.

## 1. Introduction

Benzylisoquinoline alkaloids (BIAs) are a diverse group of secondary metabolites predominantly found in plants of the Ranunculales order, particularly in the Papaveraceae family, and include pharmacologically important compounds such as morphine, codeine, papaverine, and sanguinarine (hereby referred to as morphinans) [1,2]. These compounds, derived from tyrosine, play crucial roles in plant defense and have been traditionally produced in *Papaver* species despite the complexity of their biosynthetic pathways [3,4], thus raising the question whether symbiotic plant–microbial interactions could contribute to the biosynthesis of these compounds.

One such group of symbionts, fungal endophytes, inhabit healthy plant tissue without causing visible damage or altering morphology [5,6,7]. These fungi establish prolonged residency within plants, emerging as pivotal reservoirs of bioactive compounds essential for medicinal and agricultural applications [8]. Their presence significantly augments host plant adaptability and facilitates the heightened production of bioactive metabolites [9]. Notably, certain endophytic fungi exhibit the ability to produce host-like secondary metabolites, including taxol and camptothecin [10,11]. Intentionally isolating endophytic fungi from medicinal plants is a promising approach to discover new species that produce a range of secondary metabolites with diverse biological activities [12].

Recent studies indicate that consortium inoculation boosts morphine and thebaine content in poppy plants, enhancing photosynthetic efficiency and yields [13,14]. This method influences metabolic pathways, amplifies the expression of biosynthetic genes, and augments key gene expression such as codeinone reductase (COR), crucial for morphine biosynthesis [14]. This prompted our initial investigation of endophytic fungi associated with *Papaver* species.

In our recent study of endophytic fungi associated with *Papaver* species collected from Iran and their secondary metabolite profiles for morphine alkaloids, we identified some morphinan-producing endophytic fungi [15]. The morphinan alkaloid contents in the extracts of isolated endophytic fungi were quantified using high-performance liquid chromatography (HPLC). Among the morphinan alkaloid-producing fungal isolates, IRAN 4653C exhibited the highest yield. Furthermore, morphinan production by this strain was confirmed through gas chromatography–mass spectrometry (GC-MS). Phylogenetic analyses based on ITS, TEF-1α, and TUB2 gene sequences, combined with detailed morphological characterization, revealed that IRAN 4653C represents a previously undescribed species within the genus *Pithoascus*. Accordingly, the name *Pithoascus kurdistanensis* sp. nov. strain CBS 149789 was proposed for this novel taxon [15]. We hypothesize that *P. kurdistanensis* can independently synthesize morphinan alkaloids, thereby contributing to the overall production of morphinans in *Papaver* species. If validated, this would constitute the first evidence of de novo morphinan alkaloid biosynthesis by a fungal endophyte, offering new perspectives on endophyte-mediated secondary metabolism and the evolutionary convergence of specialized metabolite pathways in plant–fungus associations.

Recent advances in next-generation sequencing have revolutionized the study of endophytic fungi [16]. The integration of long-read (e.g., Oxford Nanopore) and short-read (e.g., Illumina) technologies allows for the generation of high-quality, chromosome-level genome assemblies that overcome the challenges posed by repetitive regions and complex genomic structures [17]. Deep sequencing provides comprehensive insights into biosynthetic gene clusters and regulatory networks, which are essential for deciphering the molecular mechanisms of secondary metabolism [18]. This genomic approach is crucial for identifying candidate genes and pathways that could underpin the independent production of medicinal alkaloids by fungal endophytes.

To investigate these possibilities, we performed a complete genome sequencing and annotation of *P. kurdistanensis*. Our primary objective was to delineate the genetic landscape of this fungus and identify genes involved in isoquinoline alkaloid biosynthesis.

## 2. Materials and Methods

### 2.1. Fungus Growth Conditions

*P. kurdistanensis* strain CBS 149789 was isolated from disease-free roots of *P. bracteatum* in Kurdistan province, western Iran. Fresh cultures were initiated on potato dextrose agar (PDA) at 25 °C. Small agar plugs from these plates were then transferred to 25 mL of potato dextrose broth (PDB) in 250 mL flasks and incubated at 28 °C with shaking at 200 rpm for 10 days. Biomass from the liquid cultures was collected by centrifugation at 16,000× *g* for 5 min, and the harvested mycelia were ground to a fine powder in liquid nitrogen.

### 2.2. DNA and RNA Preparations

Genomic DNA was extracted from 200 mg of flash-frozen, pulverized *P. kurdistanensis* mycelia using the DNeasy PowerSoil Pro extraction kit (QIAGEN Canada Ltd., Toronto, ON, Canada) according to the manufacturer’s protocol. DNA yield was quantified using the Qubit dsDNA BR Assay Kit (Thermo Fisher, Waltham, MA, USA) on a Qubit 2.0 Fluorometer, and fragment size distribution was determined with a FemtoPulse system (Agilent Technologies, Santa Clara, CA, USA); see Supplementary Figure S1 for the electrophoregram.

For RNA extraction, *P. kurdistanensis* was cultured under two conditions to capture a comprehensive transcriptome. Liquid cultures were grown in 25 mL of PDB in 250 mL flasks at 200 rpm, while solid cultures were established on 10 mL of PDA in 50 mm Petri dishes overlaid with a 0.45 μm nylon membrane. All cultures were incubated at 28 °C for 10 days. Liquid culture biomass was collected by centrifugation at 16,000× *g* for 5 min, and mycelia from solid media were harvested by scraping with a sterile scalpel. The harvested material was then ground to a fine powder in liquid nitrogen. Subsequently, 1 mL of TRIzol Reagent (Thermo Fisher, Waltham, MA, USA) was added to 200–300 mg of frozen mycelial powder, followed by a 5-min incubation at 56 °C to improve cell wall lysis. Following the addition of 200 μL of chloroform and vigorous shaking for 15 s, the sample was incubated at room temperature for 3 min and then centrifuged at 13,000× *g* for 15 min at 4 °C. The lysate was transferred to a new tube and centrifuged for 2 min at full speed, after which the supernatant was mixed with an equal volume of 70% ethanol and applied to a RNeasy spin column (QIAGEN Canada Ltd., Toronto, ON, Canada) for RNA purification, following the QIAGEN RNeasy Mini Kit protocol(QIAGEN Canada Ltd., Toronto, ON, Canada). RNA quantity and integrity were assessed using the Qubit RNA BR Assay Kit (Thermo Fisher, Waltham, MA, USA) and the Agilent RNA 6000 Nano Kit (Agilent Technologies, Santa Clara, CA, USA), respectively (see Supplementary Figure S2).

### 2.3. Whole Genome Sequencing Using Illumina, Nanopore

Purified DNA samples were sequenced using the Illumina NovaSeq 6000 platform (Genome Quebec Centre of Expertise and Services, Montréal, QC, Canada) to generate paired-end reads (2 × 150 bp). In parallel, 1 µg of DNA was processed with Oxford Nanopore Technologies (ONT, Oxford, UK) native sequencing kit (SQK-LSK109), and the library was enriched for fragments ≥3 kb using ONT’s Large Fragment Buffer. Sequencing was performed on a GridION sequencer (ONT, Oxford, UK) equipped with an R9.4.1 Flow Cell. Basecalling was conducted in real time using MinKNOW v22.04 with Guppy version 6.4.6.

### 2.4. Hybrid Genome Assembly

**Figure 1:**
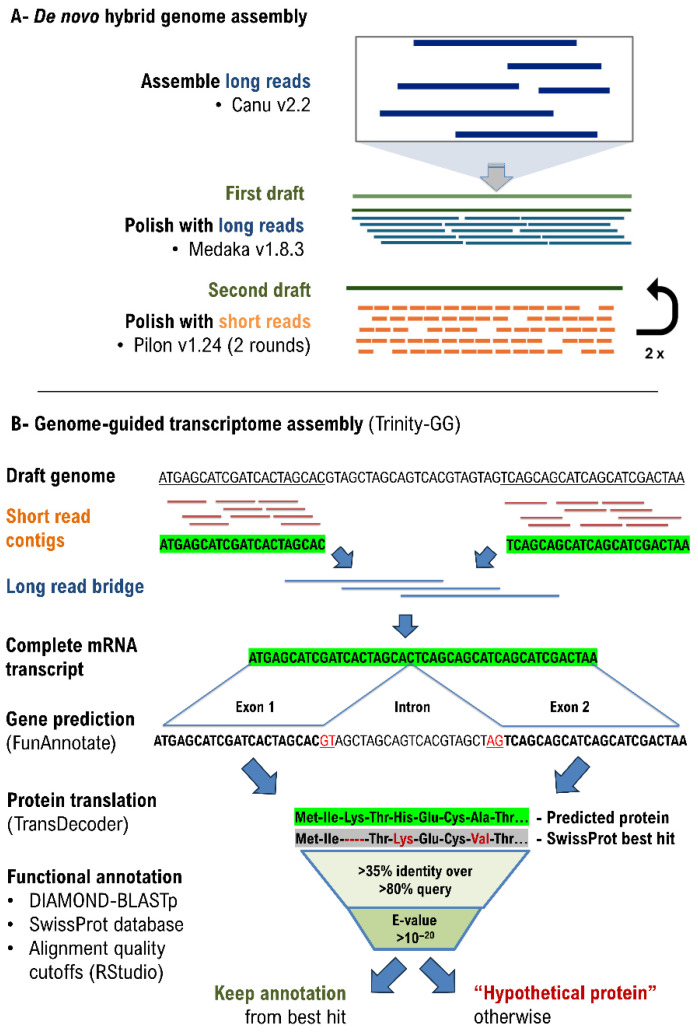
Workflow for the genome assembly and annotation of *P. kurdistanensis* CBS 149789. Briefly, a draft genome was assembled first from Nanopore long reads (with CANU) and was then corrected twice by short-read polishing to mitigate base calling errors in the first draft (with Pilon). Repeated elements were then annotated and masked to obtain a second draft, which was then annotated along with a transcriptome assembly from the same organism (with Funannotatev1.8.15).

A summarized workflow is available in Figure 1. *P. kurdistanensis* long reads were assembled first, using CANU v2.2 [19] with an estimated genome size of 34 Mbp. Before assembling, long reads were filtered and trimmed with filtlong v0.2.0 (https://github.com/rrwick/Filtlong (accessed on 1 May 2023)) to select only high-quality reads of 1 kb or above, up to a target estimated coverage of 65×. Then, short reads were aligned to the draft assembly using bwa v-0.7.17 with default parameters [20]. The resulting alignment was used to polish the draft assembly twice with Pilon v1.24 [21]. Prior to polishing, NovaSeq short reads were filtered and trimmed of TruSeq 3 adapters by Trimmomatic v0.31 with the following parameters: minimum Phred quality score of 15 over a 4 bp sliding window, and a minimum length of 36 bp.

### 2.5. Genome Completeness and Ploidy

Assembly completeness was assessed using BUSCO v5.2.2 [22] with the “sordariomycetes_odb10” dataset. Repetitive elements were identified using RepeatModeler v2.0.3 in conjunction with RepeatMasker v4.1.5 [23]. To assess whether smaller contigs (contigs 10–13) were artifactual duplications from larger contigs, sequence alignments were performed against the chromosome-sized contigs (contigs 1–9) using the “map to reference” function in Geneious Prime^®^ 2023.1.2, with minimap2 [24] run under default parameters. Genome ploidy was estimated using purge_haplotigs v1.1.3 [25], which analyzes read depth and sequence similarity to distinguish primary contigs from redundant haplotigs.

### 2.6. Gene Prediction

Gene prediction was performed with Funannotate v1.8.15 [26] using the masked genome as input and a reference-guided transcriptome as supplementary evidence to improve annotation accuracy. To generate transcriptomic data, total RNA extracted from *P. kurdistanensis* was sequenced using both short-read (Illumina Inc., San Diego, CA, USA) and long-read (ONT) platforms. For Illumina sequencing, 1 µg of total RNA was enriched for polyadenylated mRNA using the NEBNext Poly(A) mRNA Magnetic Isolation Module (New England Biolabs, Ipswich, MA, USA). cDNA libraries were prepared using the NEBNext Ultra II Directional RNA Library Prep Kit (New England Biolabs, Ipswich, MA, USA), following the manufacturer’s protocol. Library quality was assessed with an Agilent BioAnalyzer, and concentrations were determined using the Qubit dsDNA High Sensitivity Assay Kit (Thermo Fisher, Waltham, MA, USA). Sequencing was performed on an Illumina MiSeq system using V3 600-cycle chemistry (Illumina Inc., San Diego, CA, USA), yielding paired-end 2 × 300 bp reads.

For Oxford Nanopore sequencing, direct RNA sequencing was performed using the SQK-RNA002 kit (Oxford Nanopore Technologies, Oxford, United Kingdom), which allows for sequencing of native poly(A)-tailed RNA molecules without reverse transcription or second-strand synthesis. Library preparation was done following the manufacturer’s instructions, and sequencing was conducted on a GridION platform using a R9.4.1 flow cell. Basecalling was done in real time using Guppy v6.4.6 within MinKNOW 22.04 as well [27].

Long and short reads (from both liquid and solid growth conditions) were assembled together with Trinity v2.14.1 [28] in guided mode, with the draft genome assembly used as a reference.

### 2.7. Gene Product Annotation

Gene products were translated into proteins with TransDecoder v5.7.1 (https://github.com/TransDecoder/TransDecoder accessed on 10 September 2025) from the draft assembly inFASTA format and gene predictions from FunAnnotate v1.8.15 (in GFF3 format). Then, DIAMOND v2.1.12 [29] as used in “blastp” mode with parameters “—sensitive” and “—max-target-seqs 1” to assign best hits from the SwissProt database [30] to each translated gene. This primary annotation was curated by (i) keeping best hits above mid-twilight-zone alignments, i.e., 35% identity [31], (ii) keeping best hits aligned over 70% of both target and query sequences; and (iii) keeping bets hits of E-value 10^−20^ or less. Annotations that did not meet these criteria were relabeled “hypothetical protein”, while the remaining annotations were appended to the GFF3 table. All data filtering and reshaping mentioned above was done with R v4.4.1 [32] and RStudio v2024.12.0 Build 467 [33].

### 2.8. Isoquinoline Alkaloid Biosynthesis Pathway Coverage

In parallel to DIAMOND/SwissProt annotation, functional annotations (KEGG Orthology numbers and EC numbers) were assigned to predicted gene products with KoFamScan v1.3.0 [34]. Only hits with an E-value below 10^−20^ were considered. When multiple hits were found for a coding region, only the best hit was kept. The output from KoFamScan (a two-column file containing gene product IDs and KEGG Orthology numbers) was then uploaded to the Reconstruct module from KEGG Mapper webserver [35] to list the pathways where functional annotations are listed. Specifically, for the isoquinoline alkaloid biosynthesis pathway (KEGG: map00950), a visual map was generated with KEGG Mapper to highlight assignments.

### 2.9. Mitochondrial Genome Annotation

Potential mitochondrial DNA was investigated by analyzing the four smallest contigs (contigs 10–13). A cytochrome c oxidase (COX) gene screen was performed using BLAST v2.2.26 against a custom COX gene database built from NCBI-registered sequences. For contigs that showed positive matches to COX genes, mitochondrial gene annotation was performed using Prokka v1.14.6 [36] with the following parameters: Kingdom = Mitochondrial, Genus = Other, and Genetic code = Yeast mitochondria. Annotated mitochondrial genes were mapped and visualized using Proksee (https://proksee.ca; accessed on 5 November 2025) [37].

### 2.10. Genomic Phylogenetic Tree

*P. kurdistanensis* was included in a phylogenetic analysis based on conserved genes within the Sordariomycetes dataset of the BUSCO v5.2.2 pipeline, following previously described methods [22,38]. A total of 79 genomes from Sordariomycetes clades were retrieved from the NCBI database. From the 3378 single-copy orthologs identified by BUSCO, 2301 genes present in at least 90% of the genomes were retained to build a comparative matrix. Each gene’s standard amino acid translation was individually aligned using MAFFT v7.471 [39] with the parameters --auto --maxiterate 1000. The resulting alignments were concatenated with FASconCAT-G [40]. Monomorphic and low-coverage sites were removed with BMGE v1.12 [41] with default parameters, yielding a final supermatrix of 1,557,718 amino acid sites for 80 taxa (*P. kurdistanensis* CBS 149789, 78 Sordariomycetic genomes and 1 outgroup (Eurotiomycetes)). Phylogenetic inference was conducted with IQ-TREE2 v 2.1.3 [41] with 1000 ultrafast bootstrap replicates (-bb 1000) and 1000 SH-aLRT tests (-alrt 1000). The substitution model “Q.ins + F+R10” was automatically determined by IQ-TREE2’s implementation of ModelFinderPlus (-m MFP) among 1252 substitution models based on the lowest Bayesian Information Criterion (BIC) value given the data.

## 3. Results

### 3.1. Genome Characteristics

**Figure 2:**
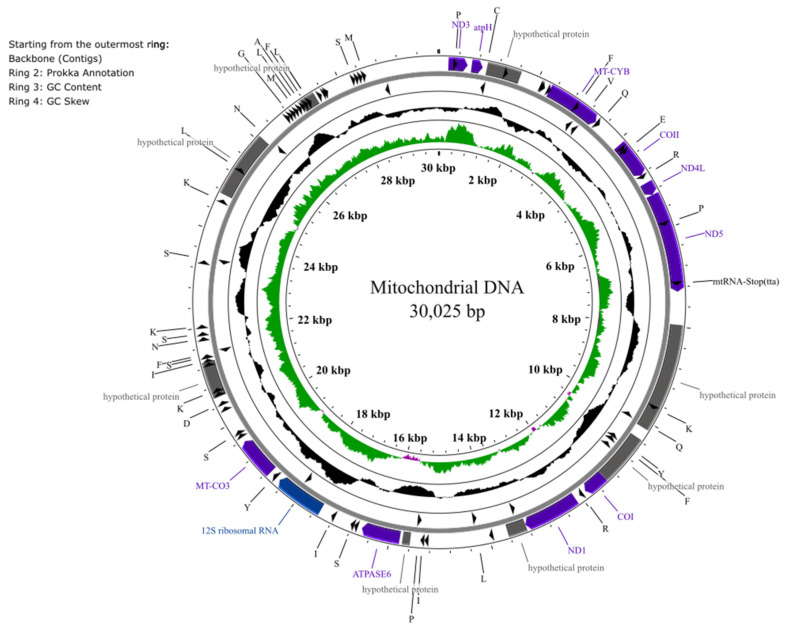
Mitochondrial genome map for *P. kurdistanensis* CBS 149789. Circular representation of the mitochondrial genome (30,025 bp) of *P. kurdistanensis*. The outermost ring displays annotated protein-coding genes (indigo), hypothetical protein genes (grey), rRNAs and tRNAs (black). Inner rings show GC content (black)and GC skew (green and purple): positive skew reflects guanine-rich regions (purple), while negative skew (green) indicates cytosine-rich regions. Annotations were generated using Prokka v1.14.6 and visualized with Proksee (https://proksee.ca; accessed on 5 November 2025).

The final assembled genome of *P. kurdistanensis* has a total size of approximately 34.0 Mb (Table 1), comprising nine chromosomal length contigs (2.61 to 6.64 Mb) and four smaller contigs (21.2 to 51.4 Kb) (Table 2). This assembly had 64.9× Nanopore coverage and 599× Illumina NovaSeq coverage. Read-depth analysis revealed a single coverage peak, indicating low heterozygosity and confirming the absence of redundant haplotigs (Supplementary Figure S4). Contig 10 (30.0 Kb), which showed significantly higher coverage (~142×), was identified as mitochondrial based on the presence of a COX gene. This contig was manually circularized with FASTA v36.5e [42] and EMBOSS extractseqv6.6.0.0 [43]. Nucleotides 30.0 kb to 51.3 kb were found to be an exact repeat of the beginning of the sequence. Prokka annotation further revealed additional conserved mitochondrial genes, including components of NADH dehydrogenase, ATP synthase, and ribosomal proteins (*rpl* and *rps*). Forty-one mitochondrial tRNA genes were also identified using Proksee’s tRNAscan-SE module (Figure 2). The mitochondrial genome of *P. kurdistanensis* is very similar to that of its close relative *Scopulariopsis brevicaulis* NC_051494.1 (28,829 bp), with 16 versus 21 CDS, 2 rRNA each, and 24 versus 22 unique tRNA genes, reflecting a largely conserved mitochondrial architecture within the Microascaceae.

Contigs 11–13 appear to be assembly artifacts. Contigs 12 and 13 aligned with regions of chromosome 4 (positions 2,972,505–2,994,980 and 1,060,760–1,078,873) with 87.2% and 85.0% pairwise identity, respectively, and included sequence gaps exceeding 3 kb. Similarly, contig 11 matched three regions of chromosome 8 within repetitive elements (positions 1,656,236–1,696,832) with 98.6% pairwise identity, suggesting redundancy or misassembly.

### 3.2. Repetitive DNA Contents

Analysis of the *P. kurdistanensis* genome revealed 12,165 repetitive elements, comprising approximately 6.37% of the total genome size (Table 3). The most prevalent categories included simple sequence repeats and low-complexity regions, together accounting for 1.20% of the genome. Long terminal repeat (LTR) retrotransposons contributed 2.15%, while DNA transposons represented 1.46%. Additionally, 1594 unclassified repetitive elements were identified, making up 1.09% of the genome. This relatively high proportion of repetitive DNA may reflect genomic plasticity and potential adaptive mechanisms related to secondary metabolism.

### 3.3. Genome Completeness

Genome completeness was evaluated using BUSCO v5.8.0 with the *sordariomycetes_odb10* dataset. The analysis identified 97.3% of expected single-copy orthologs as complete, indicating a highly complete and reliable assembly. These results support the integrity of the *P. kurdistanensis* genome and confirm its suitability for downstream functional and comparative analyses.

### 3.4. Taxonomic Assignment and Phylogenetic Analysis

**Figure 3:**
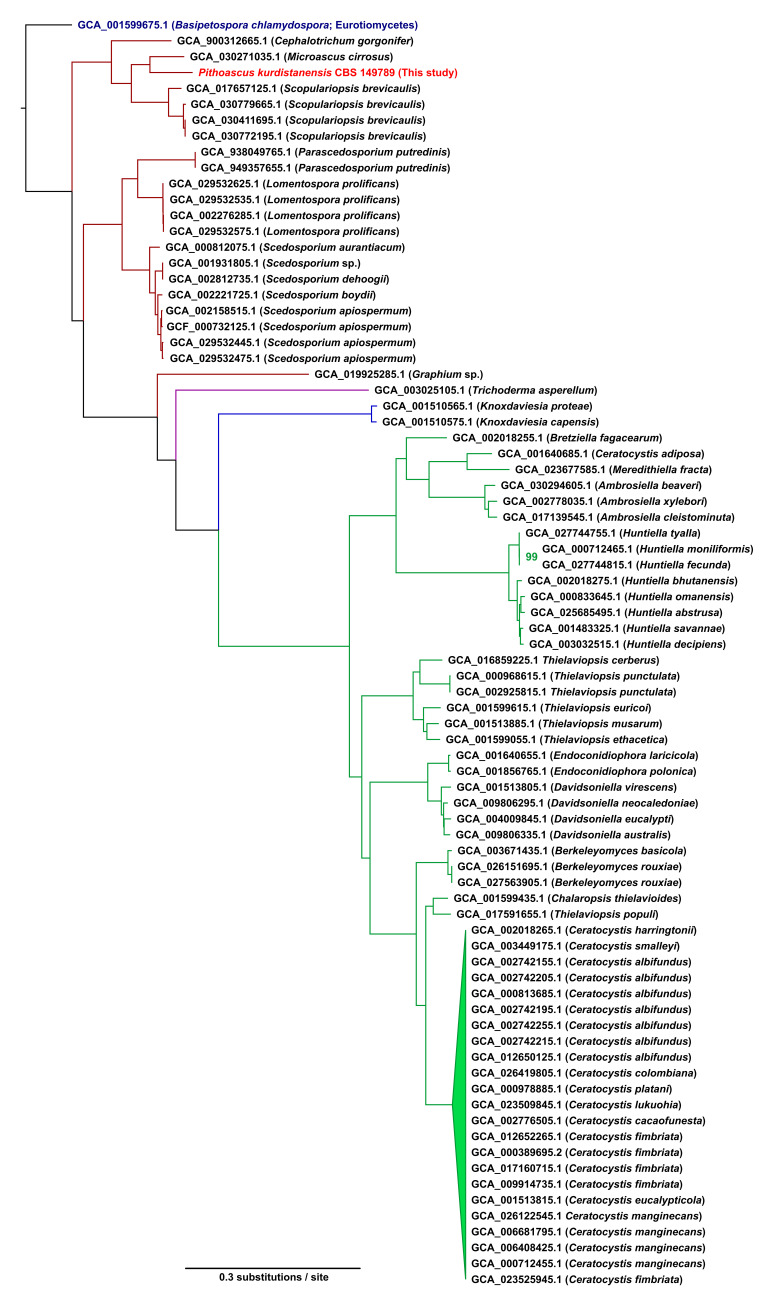
Consensus phylogenomic tree of the Sordariomycetes clade, built from universal single-copy orthologs retrieved by BUSCO. Percentages indicate node support across 1000 bootstrap trees. Branch colors indicate family assignment with standing in nomenclature as per MycoBank.org: red (Microascaceae); purple (Hypocreaceae); blue (Ophiostomataceae); and green (Ceratocystidaceae); black (non-Sordariomycete outgroup). Only bootstrap support values below 100% are shown.

Phylogenetic analysis based on conserved single-copy orthologs placed *P. kurdistanensis* within the Microascaceae family (Figure 3). The species clustered closely with *Microascus cirrosus*, forming a basal lineage within the *Scopulariopsis brevicaulis* species complex. There is strong statistical support for *P. kurdistanensis* sharing a most recent common ancestor with *Microascus cirrosus* in a basal lineage within the *Scopulariopsis brevicaulis* species complex (Microascaceae). This phylogenetic position reinforces its classification as a distinct species within the genus *Pithoascus*, consistent with previously reported morphological and molecular evidence [15].

### 3.5. Gene Prediction and Annotation

**Figure 4:**
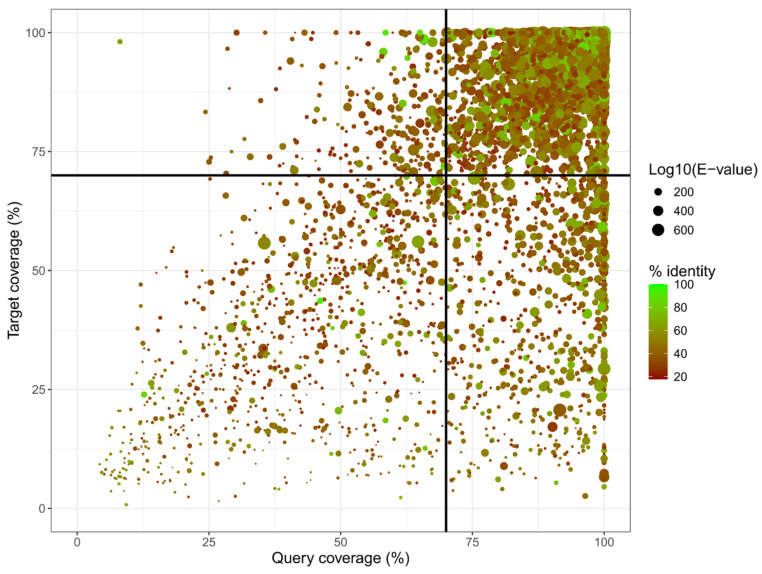
Scatter plot of *Pithoascus kurdistanensis* CBS 149789 gene annotations, grouped by alignment coverage of both queries and targets. Each dot represents a best hit by DIAMOND BLASTP v2.1.12 against the SwissProt database. The black bars, respectively, indicate the >70% query and target coverage cutoffs used to discard spurious alignments. The size of each dot is the base 10 logarithm of the E-value for each gene’s best hit. Genes that were not in the upper right quadrant (70% alignment coverage, >35% identity and E-value < 10^−20^) were labeled “hypothetical protein” to avoid false positive annotations.

A total of 8499 genes were predicted in the genome of *P. kurdistanensis*, with 6111 of them having introns. A full list of those genes, including functional annotation, is available in Supplementary Data S1. The average intron content for those genes was 2.4 ± 1.8 with a maximum of 19 introns for one single gene. There were 8324 protein-coding genes (~97.9%) in total. Of all these 8324 protein-coding genes, 5666 (68.0%) could be aligned to at least one SwissProt entry; 3709 (44.6%) shared at least 35% identity with the alignment target; and 2634 (31.6%) had aligned over 70% of either query or target sequences. Of those, 2582 protein-coding genes (31.1%) had an alignment E-value below 10^−20^, meaning that about two-thirds of proteins encoded by *P. kurdistanensis* remain uncharacterized for the sake of reliable homology-based annotation (see Figure 4).

### 3.6. Genes Potentially Involved in Alkaloid Biosynthesis

A total of 152,960 KEGG Orthology (KO) numbers were assigned to 5840 of all protein-coding genes. From those, 1177 hits over 366 genes were KO numbers linked to KEGG’s isoquinoline alkaloid biosynthesis pathway (KEGG Pathway: map00950). Only 130 of those 1177 hits had an alignment E-value below 10^−20^ for 63 protein-coding genes. For each remaining gene, only the best hit was kept as KEGG annotation. Interestingly, 52 of all 63 genes potentially involved in the isoquinoline alkaloid biosynthesis pathway were labeled “hypothetical proteins” as per the annotation discussed above. Furthermore, DIAMOND-SwissProt and KO assignments appear unrelated for the remaining 11 genes (Supplementary Table S1). Enzyme functions associated with these candidate genes (as EC numbers) were illustrated on the pathway map in Supplementary Figure S5.

## 4. Discussion

The successful assembly of a chromosome-level genome for *P. kurdistanensis* represents a significant advance in the genomic characterization of fungi within the Microascaceae family. Although hybrid sequencing approaches are becoming increasingly common [16], their application to newly described fungal genera remains limited. In this context, our use of both long-read (Oxford Nanopore) and short-read (Illumina) technologies enabled the resolution of repetitive genomic regions and structural features that might otherwise be misassembled or omitted [44]. The resulting genome quality, reflected in the high BUSCO completeness, structural continuity and haploid architecture strengthens confidence in downstream functional annotations, including the identification of secondary metabolite biosynthetic gene clusters [18].

These findings reinforce the utility of long-read sequencing in fungal genome research, particularly when studying non-model organisms with complex genomes [45]. Furthermore, the assembly serves not only as a reference for *Pithoascus* spp. but also contributes to a broader framework for understanding genomic evolution and metabolic capabilities across endophytic fungi.

The genome of *P. kurdistanensis* (34.0 Mbp) is comparable in size to its phylogenetically related species *M. cirrosus* (32.6 Mbp) and *S. brevicaulis* (32.2 Mbp), all belonging to the Microascaceae family. Indeed, there is strong phylogenomic support for *P. kurdistanensis* sharing a most recent common ancestor with *Microascus cirrosus* within the Microascaceae (Figure 3). The GC content of *P. kurdistanensis* (58.64%) falls within the range observed in *S. brevicaulis* (56.5%) and *M. cirrosus* (53.98%), reflecting shared genomic characteristics within the family [46,47]. These findings are consistent with broader fungal genomic trends, where genome sizes vary significantly, averaging 37.7 Mbp [48], with diverse chromosome numbers and ploidy levels [49]. Comparison with the mitochondrial genome of the close relative *S. brevicaulis* (NC_051494.1) reveals that it largely conserved, with comparable genome size, number of coding sequences, rRNAs, and tRNAs. Comparative analyses of these related species provide valuable insights into genome organization, evolutionary adaptations, and secondary metabolite biosynthesis within Microascaceae.

Interestingly, the BUSCO phylogenomic tree indicates a complex evolutionary history for the Microascaceae; aside for the two main branches (*Scopulariopsis*-like and *Scedosporium*-like); there is one branch that diverged from the *Scedosporidium*-like branch that includes all other fungal families (Hypocreaceae, Ophiostomataceae and Ceratocystidaceae) with *Graphium* spp. (Microascaceae) as a basal lineage. This topology also suggests that these fungal families have evolved in sequence, given their monophyletic branching in the lower branch (Figure 3). This finding is also consistent with previous multi-locus phylogenetic analysis of the Sordariomycetes based on ITS, TUB2 and EF-TU markers [15].

Some fungal genomes can comprise up to 40% repetitive sequences, although the average genome content of transposable elements (TEs) is typically around 1–4% [50]. In this study, the repetitive element content in *P. kurdistanensis* (6.37% of the total genome) is notably higher compared to other *S. brevicaulis* genomes, which contain only 1.33% of repetitive sequences [47]. These differences may reflect distinct genomic and evolutionary traits, with a higher percentage of repetitive elements potentially contributing to increased genetic variability and enhanced adaptability [44]. Moreover, the use of long-read sequencing for *P. kurdistanensis* likely contributed to a more accurate detection of these repetitive regions, whereas the short-read technology employed for *S. brevicaulis* may have underrepresented them. Although many transposable elements evolve neutrally, their occasional exaptation into regulatory elements or novel coding regions underscores their potential role as evolutionary raw material [44]. These observations suggest that the elevated repetitive content in *P. kurdistanensis* not only provides insights into its genomic architecture but may also offer a reservoir for adaptive innovation.

Biosynthesis of bioactive metabolites in endophytic fungi, particularly those metabolites that are also produced by their host plants, is a well-documented phenomenon. While some studies suggest that endophytic fungi independently develop their biosynthetic pathways, others propose genetic exchange between the fungi and their hosts, a hypothesis that warrants further exploration [51].

In our study, genomic analysis of *P. kurdistanensis* identified 63 genes potentially involved in the isoquinoline alkaloid biosynthetic pathway, as cataloged in the KEGG database. 11 of them had KO ontologies linked to tyrosine metabolism. Tyrosine serves as the primary precursor of morphinan alkaloids, and its metabolic conversion is facilitated by key enzymes such as tyrosinase, tyrosine aminotransferase, and tyrosine decarboxylase. These enzymes initiate the core steps necessary for the formation of alkaloid intermediates, providing foundational support for downstream biosynthesis [52]. To our knowledge, this is the first genome assembly for a morphinan-producing fungus. However, while these findings are promising, the pathway for morphinan alkaloid production in *P. kurdistanensis* remains unresolved, despite evidence of autonomous morphinan compound production as described in [15].

About one-third of all *P. kurdistanensis* genes could be annotated with reliable homology to SwissProt reference proteins (Figure 4). Furthermore, most genes with KEGG Orthologies for the isoquinoline alkaloid biosynthesis pathway did not have significant SwissProt best hits (Supplementary Table S1), suggesting that autonomous morphinan production by *P. kurdistanensis* could involve genes with uncharacterized molecular functions and possibly different biochemical pathways than the one of *P. bracteatum.* This is not surprising per se, as even in the post-genomics era, a significant proportion of predicted genes remain labeled as “hypothetical protein” [53]. Even the human genome, one of the most studied DNA sequences to date [54,55,56], still has more than 1000 conserved hypothetical proteins of yet unknown molecular function [57].

Note that the labeling of a protein as “hypothetical” heavily depends on the means of annotation. Indeed, homology-based annotation relies on the quality of sequences in the database. We chose SwissProt as a generic reference database because of its minimal redundancy and manually curated annotations with evidence at the experimental level [58]. This, however, could lower the number of annotated genes because of a potential lack of homologs. This choice was made to lower the chance of false positive and/or poorly characterized annotations in *P. kurdistanensis*. Therefore, a second layer of annotations (e.g., with conserved domain prediction) could be necessary to gain more information on the products of genes potentially involved in morphinan production by *P. kurdistanensis*. Such approaches include Hidden Markov models for domain identification [59], protein language models [60] and AI-based structure prediction [61].

Further studies are necessary to elucidate the underlying biosynthetic mechanisms and clarify the role of *P. kurdistanensis* in alkaloid biosynthesis. Future investigations should prioritize the functional validation of the identified genes and biochemical characterization of their enzymatic activity (such as CRISPR knockout, heterologous expression, isotope labeling). Deciphering the complete biosynthetic pathway, including potentially unique fungal modifications, will be critical to understanding the extent of fungal autonomy in morphinan production. Such insights could illuminate evolutionary parallels between plant and fungal metabolism and open promising avenues for the biotechnological exploitation of fungal endophytes as alternative platforms for sustainable alkaloid synthesis.

## 5. Conclusions

This study offers a comprehensive genomic analysis of *P. kurdistanensis*, positioning it as a promising candidate for biotechnological applications. The identification of genes related to isoquinoline alkaloid biosynthesis underscores its potential as a novel source of valuable secondary metabolites. However, the full biosynthetic pathway remains unclear, and potential metabolic exchanges with *P. bracteatum* require further investigation. The high-quality genome assembly, achieved through hybrid sequencing, provides a valuable foundation for future studies on fungal genomics and natural product biosynthesis. Functional validation of the identified genes is essential to understanding the biochemical mechanisms behind alkaloid production, opening the door for its potential use in pharmaceutical and biotechnological fields.

## Figures and Tables

**Table 1:** Summary assembly statistics for the *P. kurdistanensis* CBS 149789 genome.

	Draft (Canu)	Pilon Round #1	Pilon Round #2	Final (Masked)
Genome size (bp)	33,982,329	34,011,236	34,009,754	34,009,754
Number of contigs	13	13	13	13
Longest contig (bp)	6,631,532	6,637,200	6,636,903	6,636,903
GC %	58.61	58.64	58.64	58.64
N count *	0	0	0	2,165,484
N50 size (bp)	3,993,926	3,996,197	3,996,062	3,996,062
Contigs above N50 size	4	4	4	4
N75 size (bp)	2,860,699	2,863,255	2,863,137	2,863,137
Contigs above N75 size	6	6	6	6

* Bases masked by RepeatModeler. No undefined bases or gaps were found throughout the assembly and polishing steps, except when repeats are masked with N’s.

**Table 2:** Genomic features of *P. kurdistanensis* CBS 149789 chromosomal contigs.

Fragment	Size (bp)	CDS	rRNA	tRNA
Chromosome 1	6,636,903	1647	3	35
Chromosome 2	4,400,315	1061	1	30
Chromosome 3	4,395,346	1050	1	21
Chromosome 4	3,996,062	1041	-	18
Chromosome 5	3,330,179	798	-	17
Chromosome 6	2,863,137	643	-	10
Chromosome 7	2,828,770	682	-	9
Chromosome 8	2,813,902	656	-	14
Chromosome 9	2,608,864	714	-	9
TOTAL	33,873,478	8292	5	163

CDS: protein-coding DNA sequence. rRNA: ribosomal rRNA gene. tRNA: transfer RNA gene.

**Table 3:** Repeated element analysis for *P. kurdistanensis* CBS 149789.

	Number of Elements	Length Occupied	% of Genome Size
Retroelements	635	891,335 bp	2.62%
LINEs:	176	160,299 bp	0.47%
CRE/SLACS	57	53,664 bp	0.16%
R1/LOA/Jockey	12	1550 bp	0.00%
LTR elements:	459	731,036 bp	2.15%
Ty1/Copia	95	103,470 bp	0.30%
Gypsy/DIRS1	364	627,566 bp	1.85%
DNA transposons	523	496,324 bp	1.46%
Tc1-IS630-Pogo	315	298,983 bp	0.88%
MULE-MuDR	208	197,341 bp	0.58%
Unclassified	1594	370,488 bp	1.09%
**Total interspersed repeats:**	**4438**	**1,758,147 bp**	**5.17%**
Small RNA	22	56,043 bp	0.16%
Simple repeats	6374	282,351 bp	0.83%
Low complexity	1331	68,943 bp	0.20%
**Total bases masked:**	**12** **,** **165**	**2,165,484 bp**	**6.37%**

## Data Availability

Genome assembly and raw sequence data were deposited in the NCBI Genome and Sequence Read Archive databases under BioProject PRJNA1298848. Genome data is available under the following accession number: JBPXNJ000000000.

## Supplementary Materials

The following supporting information can be downloaded at: https://www.mdpi.com/article/10.3390/jof11120861/s1. Figure S1: FemtoPulse pulsed-field electrophoregram detailing fragment length distribution within the DNA extract of *P. kurdistanensis* CBS 149789 used for sequencing.; Figure S2: The quality of RNA extracted from *P. kurdistanensis* grown in different culture conditions; Figure S3: Full workflow for the genome assembly and annotation of *P. kurdistanensis* CBS 149789; Figure S4: Read-depth histogram for the assembled genome of *P. kurdistanensis* CBS 149789; Figure S5: *P. kurdistanensis* CBS 149789 genes involved in the isoquinoline alkaloid biosynthesis pathway; Table S1: Genes potentially involved in the isoquinoline alkaloid biosynthesis pathway.; Data S1: DIAMOND-SwissProt *ab initio* gene predictions for *P. kurdistanensis* CBS 149789 in GFF3 format.
