# Supplementary material for: Complete Genome Sequence of Pithoascus kurdistanensis CBS 149789, an Endophytic Fungus Isolated from Papaver bracteatum
Source: J Fungi (Basel). 2025 Dec 5;11(12):861. doi: 10.3390/jof11120861 (PMC12733594; doi:10.3390/jof11120861)
Supplement: Supplementary file 1 [file jof-11-00861-s001.zip › jof-3999984-supplementary.pdf]

Complete Genome Sequence of *Pithoascus kurdistanensis* CBS 149789, an Endophytic Fungus Isolated from *Papaver bracteatum*

Supplementary Materials

Sima Mohammadi [1], Jeff Gauthier [1], Guillaume Quang Henri Nguyen [1], Antony T. Vincent [1,2], Bahman Bahramnejad [3], Roger C. Levesque [1,*]

1. Institut de biologie intégrative et des systèmes, Université Laval, Quebec City, QC G1V 0A6, Canada; sima.mohammadi.1@ulaval.ca (S.M.); jeff.gauthier.1@ulaval.ca (J.G.); guillaume.quang-henri.nguyen.1@ulaval.ca (G.Q.H.N.); antony.vincent@fsaa.ulaval.ca (A.T.V.)
2. Faculté des Sciences de l’Agriculture et de l’Alimentation, Université Laval, Quebec City, QC G1V 0A6, Canada
3. Department of Plant Production and Genetics, Faculty of Agriculture, University of Kurdistan, Sanandaj 6617715175, Iran; b.bahramnejad@uok.ac.ir

* Correspondence: rclevesq@ibis.ulaval.ca

Supplementary Figures

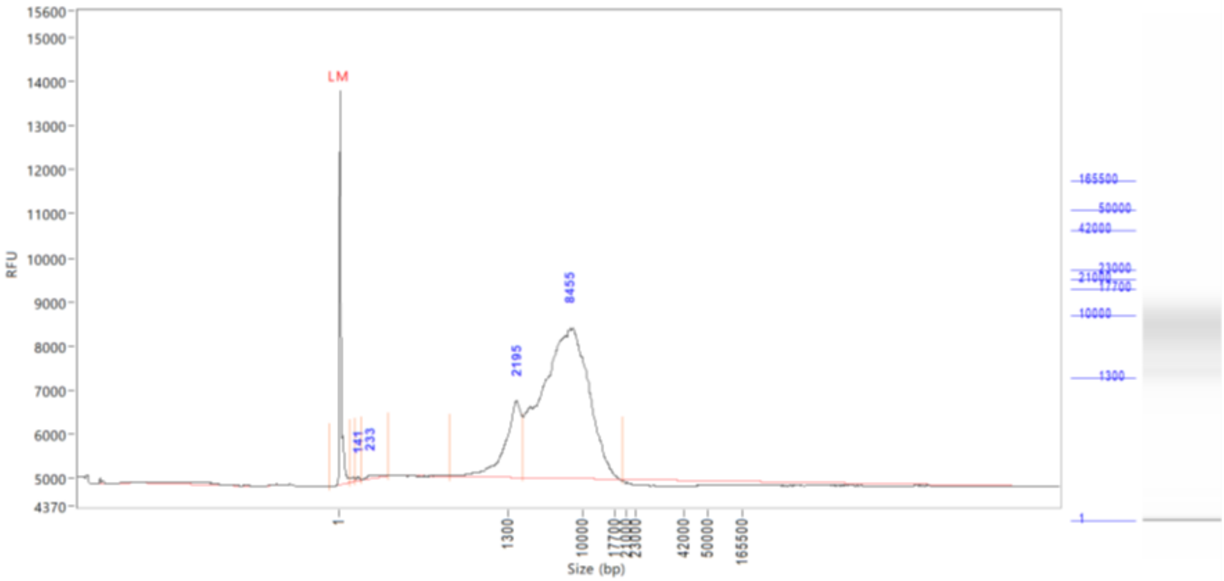

Suppl. Figure S1. FemtoPulse pulsed-field electrophoregram detailing fragment length distribution within the DNA extract of *P. kurdistanensis* CBS 149789 used for sequencing.

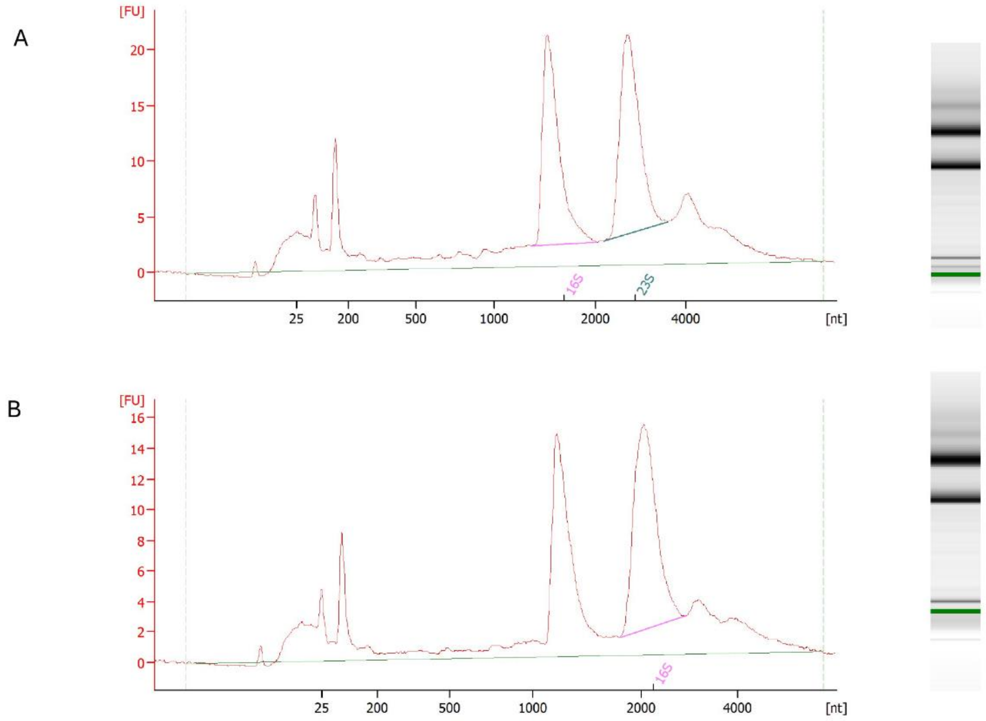

Suppl. Figure S2. The quality of RNA extracted from *P. kurdistanensis* grown in different culture conditions. BioAnalyzer profiles of total RNA isolated from cultures grown on (A) solid medium and (B) liquid medium. Clear 18S and 28S rRNA bands are visible in both conditions, indicating high RNA integrity suitable for downstream transcriptomic analyses.

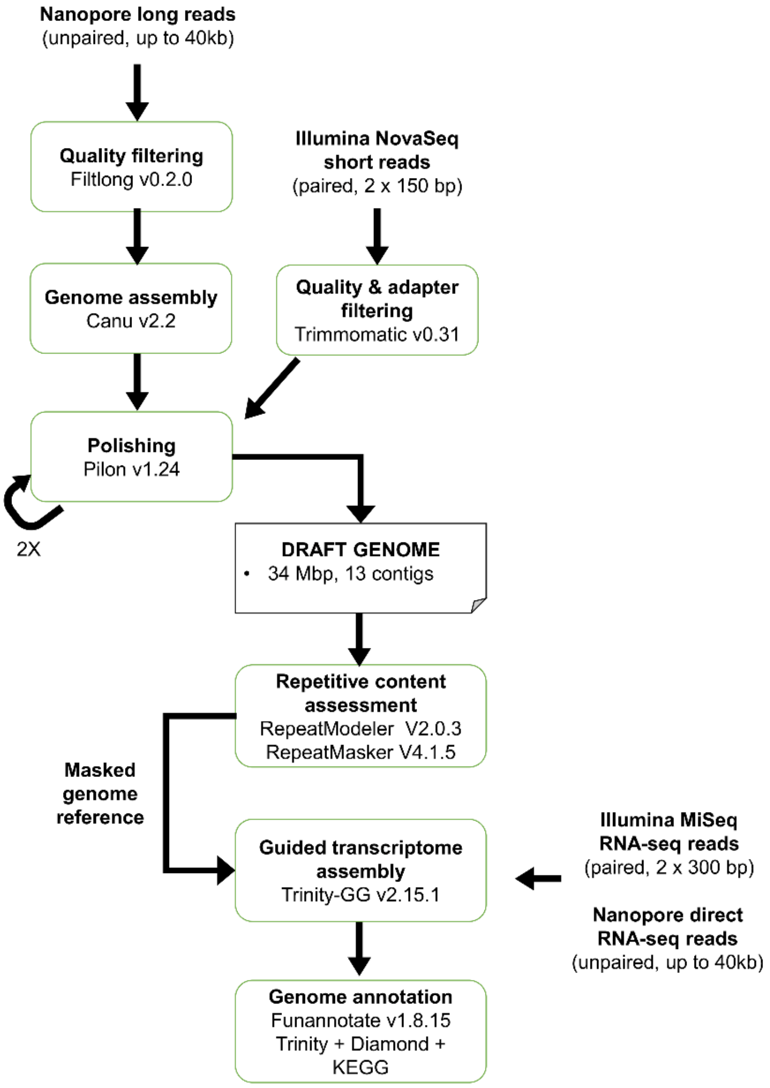

Suppl. Figure S3. Full workflow for the genome assembly and annotation of *P. kurdistanensis* CBS 149789. Briefly, a draft genome was assembled first from Nanopore long reads (with CANU) and was then corrected twice by short-read polishing to mitigate base calling errors in the first draft (with Pilon). Repeated elements were then annotated and masked to obtain a second draft, which was then annotated along with a transcriptome assembly from the same organism (with Funannotate).

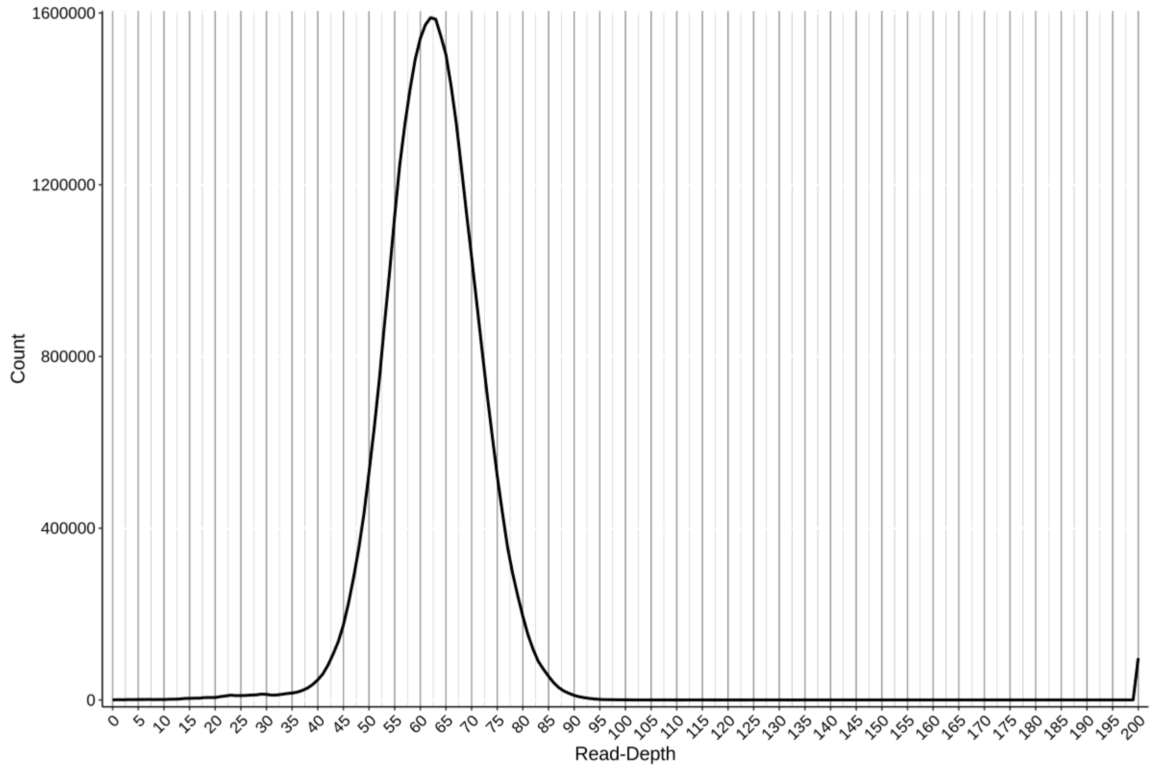

Suppl. Figure S4. Read-depth histogram for the assembled genome of *P. kurdistanensis CBS 149789*. The single coverage peak indicates a haploid genome structure with low heterozygosity and the absence of redundant haplotigs.

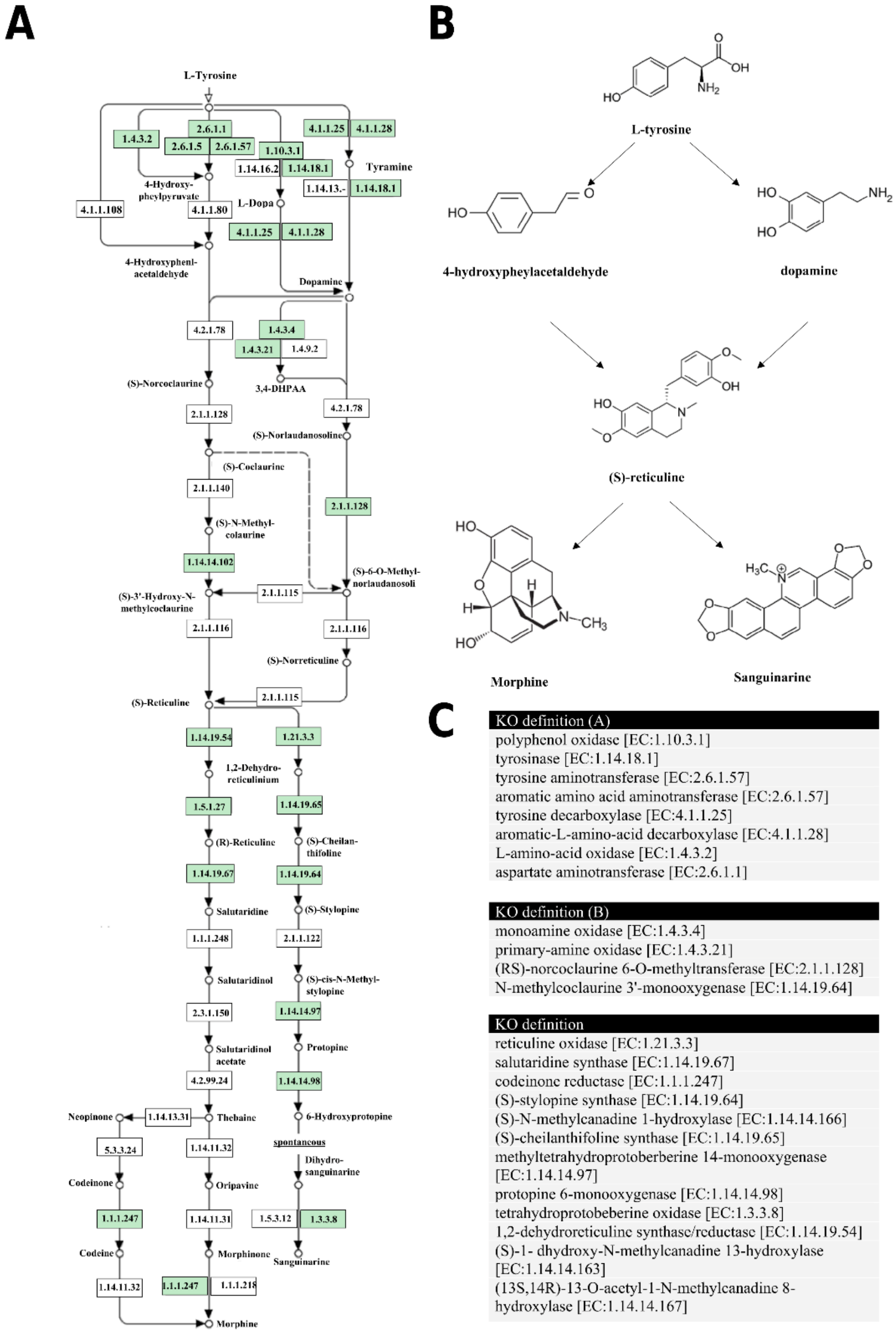

Suppl. Figure S5. ***P. kurdistanensis*** **CBS 149789 genes involved in the isoquinoline alkaloid biosynthesis pathway**. A: Pathway map from the KEGG (Kyoto Encyclopedia of Genes and Genomes). Green-labeled nodes indicate gene evidence for a given function (with EC number in label). B: Simplified overview of the isoquinoline alkaloid biosynthesis pathway from KEGG. C: Enzymes involved in specific parts of the pathway shown in B.

Supplementary Tables

Suppl. Table S1. Genes potentially involved in the isoquinoline alkaloid biosynthesis pathway. Generic annotations with DIAMOND-BLASTP (SwissProt) are also included alongside KoFamScan best hits. KO: KEGG Ontology number. DMND: DIAMOND-BLASTP best hit annotation (see Methods).

Gene_ID	*Contig*	*Start*	*End*	*DMND_annotation*	*DMND_evalue*	*KO_number*	*KO_definition*	*KO_evalue*
FUN_000010-T1	scaffold_1	68726	70230	GH30 family xylanase	8.1E-181	K00505	tyrosinase [EC:1.14.18.1]	6.9E-26
FUN_000074-T1	scaffold_1	319789	320533	hypothetical protein		K13394	codeinone reductase [EC:1.1.1.247]	2.3E-39
FUN_000102-T1	scaffold_1	404489	405172	hypothetical protein		K00505	tyrosinase [EC:1.14.18.1]	1.7E-65
FUN_000110-T1	scaffold_1	442564	443148	Ribosome biogenesis regulatory protein homolog	2.99E-64	K13385	N-methylcoclaurine 3'-monooxygenase [EC:1.14.14.102]	9.1E-24
FUN_000138-T1	scaffold_1	549406	549870	hypothetical protein		K21692	methyltetrahydroprotoberberine 14-monooxygenase [EC:1.14.14.97]	4E-21
FUN_000849-T1	scaffold_1	3422155	3423333	hypothetical protein		K00276	primary-amine oxidase [EC:1.4.3.21]	6.9E-124
FUN_000856-T1	scaffold_1	3441339	3442486	hypothetical protein		K22089	tetrahydroprotoberberine oxidase [EC:1.3.3.8]	1.8E-28
FUN_000899-T1	scaffold_1	3615173	3615930	hypothetical protein		K22096	(13S,14R)-13-O-acetyl-1-hydroxy-N-methylcanadine 8-hydroxylase [EC:1.14.14.167]	8.3E-29
FUN_001084-T1	scaffold_1	4202704	4206054	hypothetical protein		K01593	aromatic-L-amino-acid/L-tryptophan decarboxylase [EC:4.1.1.28 4.1.1.105]	1.6E-77
FUN_001290-T1	scaffold_1	4922321	4926101	hypothetical protein		K03334	L-amino-acid oxidase [EC:1.4.3.2]	2.3E-31
FUN_001452-T1	scaffold_1	5613656	5614913	hypothetical protein		K13394	codeinone reductase [EC:1.1.1.247]	1.5E-22
FUN_001462-T1	scaffold_1	5669813	5671675	hypothetical protein		K22089	tetrahydroprotoberberine oxidase [EC:1.3.3.8]	4.5E-23
FUN_001509-T1	scaffold_1	5851216	5852403	hypothetical protein		K13394	codeinone reductase [EC:1.1.1.247]	2.2E-35
FUN_001514-T1	scaffold_1	5863574	5863941	hypothetical protein		K13385	N-methylcoclaurine 3'-monooxygenase [EC:1.14.14.102]	3.4E-27
FUN_001534-T1	scaffold_1	5961609	5962876	hypothetical protein		K00274	monoamine oxidase [EC:1.4.3.4]	7.1E-137

FUN_002119-T1	scaffold_2	1689444	1692189	hypothetical protein		K22096	(13S,14R)-13-O-acetyl-1-hydroxy-N-methylcanadine 8-hydroxylase [EC:1.14.14.167]	3.5E-22
FUN_002232-T1	scaffold_2	2232321	2232545	hypothetical protein		K00505	tyrosinase [EC:1.14.18.1]	1.9E-60
FUN_002278-T1	scaffold_2	2395086	2395935	Lytic polysaccharide monooxygenase-like protein ham-7	5.86E-92	K22089	tetrahydroprotoberberine oxidase [EC:1.3.3.8]	1.7E-26
FUN_002380-T1	scaffold_2	2820049	2820840	Monothiol glutaredoxin-4	1.1E-55	K00276	primary-amine oxidase [EC:1.4.3.21]	9.8E-197
FUN_002533-T1	scaffold_2	3467761	3471420	hypothetical protein		K21692	methyltetrahydroprotoberberine 14-monooxygenase [EC:1.14.14.97]	2E-23
FUN_002631-T1	scaffold_2	3845459	3846202	hypothetical protein		K01593	aromatic-L-amino-acid/L-tryptophan decarboxylase [EC:4.1.1.28 4.1.1.105]	4.5E-24
FUN_002977-T1	scaffold_3	699438	700508	hypothetical protein		K00274	monoamine oxidase [EC:1.4.3.4]	2E-28
FUN_003096-T1	scaffold_3	1255773	1256357	Large ribosomal subunit protein uL11m	5.77E-77	K21692	methyltetrahydroprotoberberine 14-monooxygenase [EC:1.14.14.97]	2.3E-22
FUN_003330-T1	scaffold_3	2220012	2221621	hypothetical protein		K03334	L-amino-acid oxidase [EC:1.4.3.2]	3.1E-52
FUN_003636-T1	scaffold_3	3391317	3393134	hypothetical protein		K21692	methyltetrahydroprotoberberine 14-monooxygenase [EC:1.14.14.97]	1.8E-21
FUN_003656-T1	scaffold_3	3448337	3449266	hypothetical protein		K13394	codeinone reductase [EC:1.1.1.247]	2.1E-48
FUN_003721-T1	scaffold_3	3631286	3631498	hypothetical protein		K00274	monoamine oxidase [EC:1.4.3.4]	1.6E-21
FUN_003759-T1	scaffold_3	3757856	3758203	hypothetical protein		K14454	aspartate aminotransferase, cytoplasmic [EC:2.6.1.1]	3.4E-203
FUN_003783-T1	scaffold_3	3833981	3834259	hypothetical protein		K00838	aromatic amino acid aminotransferase I / 2-aminoadipate transaminase [EC:2.6.1.57 2.6.1.39 2.6.1.27 2.6.1.5]	1.6E-27
FUN_003942-T1	scaffold_4	480963	481883	hypothetical protein		K15849	bifunctional aspartate aminotransferase and glutamate/aspartate-prephenate aminotransferase [EC:2.6.1.1 2.6.1.78 2.6.1.79]	3.9E-51
FUN_004076-T1	scaffold_4	941857	942870	hypothetical protein		K13395	(S)-stylopine/(S)-canadine/(S)-nandinine synthase [EC:1.14.19.64 1.14.19.68 1.14.19.73]	1.3E-21
FUN_004112-T1	scaffold_4	1048383	1050073	hypothetical protein		K21693	protopine 6-monooxygenase [EC:1.14.14.98]	1.1E-26

FUN_004114-T1	scaffold_4	1052706	1053527	hypothetical protein		K13385	N-methylcoclaurine 3'-monooxygenase [EC:1.14.14.102]	1.7E-22
FUN_004126-T1	scaffold_4	1077741	1078028	hypothetical protein		K13395	(S)-stylopine/(S)-canadine/(S)-nandinine synthase [EC:1.14.19.64 1.14.19.68 1.14.19.73]	2.6E-23
FUN_004161-T1	scaffold_4	1216470	1217249	Disulfide-bond oxidoreductase YfcG	4.26E-50	K13385	N-methylcoclaurine 3'-monooxygenase [EC:1.14.14.102]	1.7E-22
FUN_004228-T1	scaffold_4	1425945	1426992	hypothetical protein		K13385	N-methylcoclaurine 3'-monooxygenase [EC:1.14.14.102]	7.5E-27
FUN_004281-T1	scaffold_4	1635849	1636268	hypothetical protein		K13395	(S)-stylopine/(S)-canadine/(S)-nandinine synthase [EC:1.14.19.64 1.14.19.68 1.14.19.73]	6.2E-22
FUN_004377-T1	scaffold_4	1981040	1981633	hypothetical protein		K13394	codeinone reductase [EC:1.1.1.247]	6.5E-40
FUN_004385-T1	scaffold_4	2010378	2011257	Large ribosomal subunit protein uL16m	1.95E-100	K00276	primary-amine oxidase [EC:1.4.3.21]	6.7E-214
FUN_004460-T1	scaffold_4	2328774	2330048	hypothetical protein		K13395	(S)-stylopine/(S)-canadine/(S)-nandinine synthase [EC:1.14.19.64 1.14.19.68 1.14.19.73]	6E-32
FUN_004779-T1	scaffold_4	3523092	3524156	hypothetical protein		K00505	tyrosinase [EC:1.14.18.1]	3.2E-49
FUN_005052-T1	scaffold_5	615194	615793	hypothetical protein		K21693	protopine 6-monooxygenase [EC:1.14.14.98]	2E-26
FUN_005087-T1	scaffold_5	743533	745554	hypothetical protein		K00307	reticuline oxidase [EC:1.21.3.3]	5.9E-32
FUN_005282-T1	scaffold_5	1626957	1630070	Uncharacterized protein YMR196W	0	K00838	aromatic amino acid aminotransferase I / 2-aminoadipate transaminase [EC:2.6.1.57 2.6.1.39 2.6.1.27 2.6.1.5]	2E-133
FUN_005412-T1	scaffold_5	2097712	2100867	hypothetical protein		K21693	protopine 6-monooxygenase [EC:1.14.14.98]	2.7E-23
FUN_005425-T1	scaffold_5	2155891	2159919	hypothetical protein		K00505	tyrosinase [EC:1.14.18.1]	1.1E-64
FUN_005502-T1	scaffold_5	2436453	2436809	hypothetical protein		K13394	codeinone reductase [EC:1.1.1.247]	1.1E-41
FUN_005627-T1	scaffold_5	2915971	2919116	Vacuolar protein 8	4.22E-154	K14455	aspartate aminotransferase, mitochondrial [EC:2.6.1.1]	7.1E-230
FUN_005710-T1	scaffold_5	3220434	3221773	Xaa-Arg dipeptidase	1.84E-78	K00505	tyrosinase [EC:1.14.18.1]	1.2E-76
FUN_006108-T1	scaffold_6	1624765	1626218	hypothetical protein		K00505	tyrosinase [EC:1.14.18.1]	3.3E-44
FUN_006202-T1	scaffold_6	2038012	2039424	hypothetical protein		K13394	codeinone reductase [EC:1.1.1.247]	1.5E-40

FUN_006230-T1	scaffold_6	2265706	2266194	hypothetical protein		K22096	(13S,14R)-13-O-acetyl-1-hydroxy-N-methylcanadine 8-hydroxylase [EC:1.14.14.167]	1E-22
FUN_006269-T1	scaffold_6	2480355	2480804	hypothetical protein		K13394	codeinone reductase [EC:1.1.1.247]	1.3E-38
FUN_006282-T1	scaffold_6	2517023	2517976	hypothetical protein		K00505	tyrosinase [EC:1.14.18.1]	1E-26
FUN_006298-T1	scaffold_6	2550287	2552152	hypothetical protein		K00815	tyrosine aminotransferase [EC:2.6.1.5]	6.1E-25
FUN_006340-T1	scaffold_6	2728114	2729543	hypothetical protein		K00815	tyrosine aminotransferase [EC:2.6.1.5]	2.1E-36
FUN_006466-T1	scaffold_7	357897	359340	hypothetical protein		K13394	codeinone reductase [EC:1.1.1.247]	4E-42
FUN_006879-T1	scaffold_7	2066790	2067503	hypothetical protein		K00505	tyrosinase [EC:1.14.18.1]	1.7E-49
FUN_006894-T1	scaffold_7	2105984	2106244	hypothetical protein		K13385	N-methylcoclaurine 3'-monooxygenase [EC:1.14.14.102]	1.3E-23
FUN_007056-T1	scaffold_7	2754788	2755297	hypothetical protein		K13394	codeinone reductase [EC:1.1.1.247]	2.1E-41
FUN_007256-T1	scaffold_8	635736	637022	hypothetical protein		K13394	codeinone reductase [EC:1.1.1.247]	3.9E-39
FUN_007334-T1	scaffold_8	972377	972978	Ubiquitin-conjugating enzyme E2-20 kDa	4.72E-49	K13383	(RS)-norcoclaurine 6-O-methyltransferase [EC:2.1.1.128]	2.1E-22
FUN_007341-T1	scaffold_8	991946	992416	hypothetical protein		K13394	codeinone reductase [EC:1.1.1.247]	2.7E-36
